# Biological and mechanical challenges in the endodontic treatment of immature teeth with pulp necrosis: insights based on a Series of Atypical Clinical Cases

**DOI:** 10.2340/biid.v12.43427

**Published:** 2025-04-04

**Authors:** Julian G. Leprince, Motoki Okamoto, Matthias Widbiller, Julien Beauquis, Simon Mariano Pedano, Kerstin M. Galler, Yusuke Takahashi

**Affiliations:** a Division of Cariology and Endodontology, University Clinics of Dental Medicine (CUMD), University of Geneva, 1211 Geneva, Switzerland; b Department of Oral Science and Translational Research, College of Dental Medicine, Nova Southeastern University, Fort Lauderdale, FL, USA; c Department of Conservative Dentistry and Periodontology, University Hospital Regensburg, Regensburg, Germany; d Department of Adult and Child Dentistry, Cliniques Universitaires Saint-Luc, Brussels, Belgium; e Department of Oral Health Sciences, Endodontology, University Hospitals Leuven, KU Leuven, Leuven, Belgium; f Department of Operative Dentistry and Periodontology, Friedrich-Alexander-University Erlangen-Nuernberg, Erlangen, Germany; g Department of Restorative Dentistry and Endodontology, Osaka University Graduate School of Dentistry, Suita, Osaka, Japan

**Keywords:** Dental pulp, endodontics, revitalisation, regeneration, biocompatible materials, apexification

## Abstract

Over the past two decades, dental pulp regeneration has become a major focus in endodontology. The currently applied clinical strategies are referred to as ‘revitalisation’ procedures. These biology-based treatment strategies aim at regenerating lost pulp tissues in necrotic teeth, in the absence or even more in the presence of periapical bone lesion, clinical signs and symptoms. Such approaches are generally – but not exclusively – used in immature teeth to promote root maturation, both in length and in thickness, ultimately to reduce their risk of fracture. A growing body of evidence has led to increased understanding and reliability of these treatment strategies, which are now considered as a valid alternative treatment option besides conventional ones, mainly the apical plug technique. However, all systematic reviews evaluating clinical outcomes concluded that there is a lack of robust long-term studies on the subject; most published cases of revitalisation having a relatively short-term follow-up, usually under 2 years. In this context, several major challenges remain to be addressed to better understand the promises and limitations of revitalisation procedures as compared to other treatment options, mainly the placement of an apical plug made of hydraulic calcium silicate cement. The purpose of this paper was therefore to identify some of the important remaining challenges related to such procedures, which can be broadly categorised into biological and mechanical ones, affecting treatment success and tooth survival. Meeting these challenges requires close collaboration between both researchers and clinicians, to establish guidelines, evaluate and understand treatment outcomes, and update guidelines accordingly. However, it is not always easy for researchers to understand the clinical reality faced by practitioners. In order to facilitate their mutual understanding, the aforementioned challenges were illustrated by providing clinical context through a series of atypical clinical cases with long-term follow-up (4–8 years).

## Introduction

Dental biomaterials research is evolving similarly to other areas in the biomaterials field [1], with a trend moving away from purely restorative treatments and increasingly focussing on regenerative approaches. This evolution generates new needs for all sorts of materials that are expected to be bioactive, biodegradable, biomimetic, antibacterial, injectable or printable, and possibly combined with drug delivery characteristics [2–4], all of which represent new challenges for biomaterials researchers in the field of regenerative dental medicine. It is therefore essential for biomaterials researchers to be aware of the challenges faced by clinicians, to better address them in the future.

One of those challenges is the regeneration of the lost dental pulp tissues in necrotic teeth, which has become a major focus in endodontics over the past two decades.

According to the recent S3-level clinical practice guidelines by the European Society of Endodontology (ESE), regeneration is now considered as one of the recommended approaches to consider for the treatment of immature teeth with pulp necrosis either with or without apical periodontitis [5]. The regenerative strategies currently applied clinically for this purpose are referred to as ‘revitalisation’ procedures. These have been described as therapies aimed at ‘the (re)generation of pulp-like tissue inside the root canal after inducing an influx of stem cells from the apical papilla’ [6].

These biology-based treatment strategies are particularly used in immature teeth to promote the maturation of the root, both in length and in thickness, ultimately to reduce their risk of fracture to extend tooth longevity. A growing body of evidence has led to increased understanding and reliability of these revitalisation treatment strategies, which are now considered for several years as being part of the endodontic treatment spectrum of immature teeth, not only by the ESE [6, 7] but also by the American Association of Endodontists (AAE) [8], and the European Academy of Paediatric Dentistry (EAPD) [9]. It is now recommended to clinicians as a valid alternative treatment option alongside the conventional approach, which primarily involves the placement of an apical plug made of hydraulic calcium silicate cement [5]. However, many uncertainties remain regarding revitalisation procedures. This is notably illustrated by the low predictability repeatedly deplored regarding the completion of root formation [10–12]. In addition, apart from some rare cases [13], no actual regeneration of pulp-like tissue has been reported in the vast majority of clinical reports and studies. In the latter, the tissues found in the canal were a combination of periodontal ligament, cementum, and bone-like tissue in various proportions, which can be described as repair rather than regeneration [14].

A substantial number of recent reviews are available on the subject of revitalisation, including narrative, scoping, or systematic reviews and meta-analysis [9–12, 15–23]. Among the clinical data available, the vast majority are limited to case reports or case series [11, 19]. While the ESE recommendations for conventional root canal treatments are to perform a yearly follow-up for at least 4 years to evaluate healing [24], most published cases of revitalisation have a relatively short-term follow-up, usually under 2 years, rarely over 4 years [11, 15, 17, 23]. All the available systematic reviews evaluating clinical outcomes conclude to a paucity of robust long-term studies (at least beyond 1 year) on the subject [11, 18, 21]. For example, Meschi et al. reported only five clinical trials meeting their inclusion criteria, with minimum 10 teeth/arm and 1-year follow-up [18]. Therefore, several major challenges remain to be addressed to better understand the promises and limitations of revitalisation procedures.

The purpose of this paper was thus to identify some of the important remaining biological and mechanical challenges related to revitalisation procedures. Researchers and clinicians both have significant roles to play in this venture, but it is not always easy for the former to understand the clinical reality faced by the latter, making it difficult to contribute in a relevant manner. Hence, this work also intends to illustrate the aforementioned challenges by providing clinical context through a series of atypical clinical cases with long-term follow-up.

## Case series

The present case reports have been described according to Case Report (CARE) guidelines [25]. Informed consent was obtained from each patient after the treatment for using the data in a case report publication.

### Case #1: Successful revitalisation procedure of dens evaginatus with an abnormal root defect (Y.T.)

**Figure 1:**
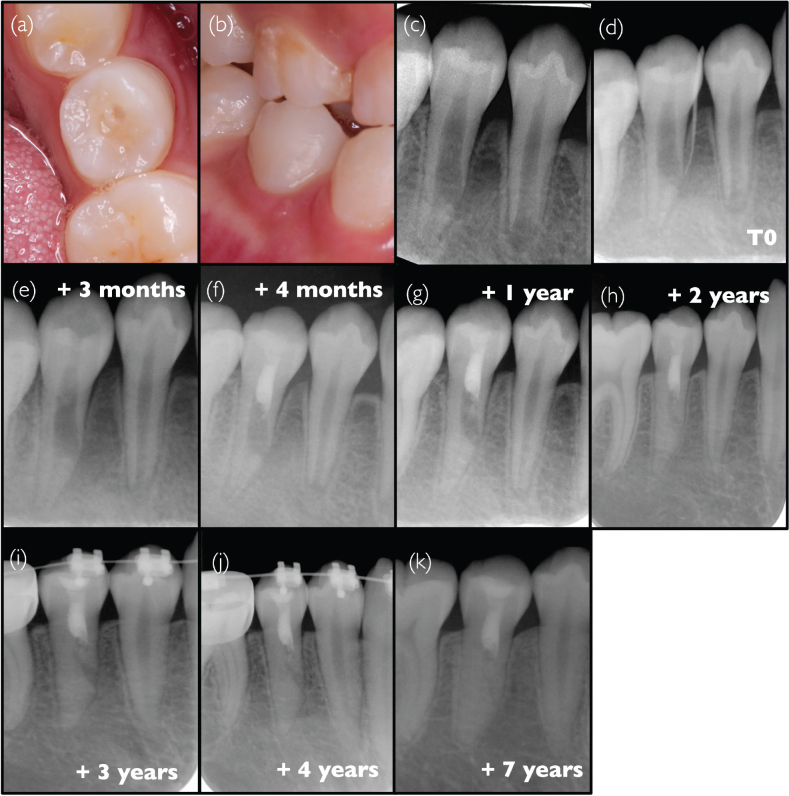
Clinical case #1 (A and B) Pre-operative intraoral photographs and (C) pre-operative radiograph. (D) pre-operative radiograph showing a gutta-percha point inserted into the sinus tract. (E) Radiograph demonstrating the situation after the application of a calcium hydroxide medication. (F) Radiograph following the completion of the revitalisation procedure. (G–K) Follow-up radiographs taken at 1 year and up to 7 years after the initial visit.

A 10-year-old Asian male patient was referred to the clinical Department of Restorative Dentistry and Endodontics (Osaka University Dental Hospital, Osaka, Japan) after the identification of a problem in the lower right mandibular premolar region 1 week before; the patient did not have any symptoms. The patient had no history of trauma or prior medication, no specific general medical or family history was noted. Fissure sealant had been applied to the occlusal surface of the second premolar, but a fracture of the central cusp was observed (Figure 1A). An intraoral examination revealed a slight swelling close to the cervical areal of the same tooth (Figure 1B). The tooth responded slightly painfully to percussion. Pulp sensibility tests, including cold stimulation test (Pulper, GC, Japan) and electrical pulp test (Dentotest model TB09, Perkell Products, USA), demonstrated no response compared to adjacent teeth. Periodontal tests were within normal limits, including periodontal probing depths < 3 mm, and without any abnormal tooth mobility. An intraoral radiograph revealed that tooth #45 presented an immature apex and an irregular root canal shape with a root defect on the mesial side, with some uncertainty regarding the presence of lateral bone radiolucency (Figure 1C). Analysis of clinical and radiographic findings led to the suspicion of chronic apical periodontitis, secondary to pulp infection due to the fracture of the central cusp in a case of dens evaginatus. However, because of the atypical nature of the case combined with the uncertainty of radiograph interpretation, it was decided to monitor until the next follow-up examination scheduled 4 months later. At the second visit, however, a sinus tract appeared, without any changes in symptoms compared to the first visit. A new radiograph was taken after insertion of a gutta percha cone in the sinus tract, pointing to the lateral root defect as the origin of the sinus tract (Figure 1D), hence confirming the diagnosis, chronic apical abscess. It was hypothesised that the unusual aspect of the root was on account of the presence of a terminal root bifurcation, where development stopped in only one of the two root ends due to tooth necrosis. It was decided to perform a revitalisation procedure to achieve additional root development after elimination of the root canal infection. Under rubber dam isolation, the root canal was accessed without local anaesthesia using a diamond bur and Peeso reamers, and root canal irrigation was performed with 2.5% NaOCl (sodium hypochlorite) and 3% EDTA (ethylenediaminetetraacetic acid) (Smear Clean, Nishika, Japan), with the assistance of an ultrasonic device (Suprasson P-Max, France); almost no pain was reported by the patient. Calcium hydroxide was applied in the root canal, and the cavity was temporarily sealed with glass ionomer cement (GIC) (Base Cement, Shofu, Japan). At the third visit, 3 months later, the patient presented no symptoms, and the sinus tract and swelling had disappeared. A new radiograph revealed little change from the previous visit (Figure 1E). After irrigation with EDTA, calcium hydroxide was placed, and the cavity was sealed with GIC. After 1 month, the patient claimed that the percussion pain had completely disappeared. Following root canal irrigation using an EDTA solution and drying with paper cones, bleeding was induced by instrumenting with a #15 K-file beyond the apical foramen. After confirmation of haemostasis, a gelatine sponge (Spongel, Astellas, Japan) was inserted into the middle portion of the root canal, and ProRoot MTA (Dentsply Sirona, Switzerland) was placed on the gelatine sponge, followed by a GIC temporary coronal restoration (Figure 1F). At this stage, the irregularly shaped defect on the mesial side appeared slightly smaller compared to that at the first visit. A gradual decrease of the irregular defect could be noticed on the successive radiographs, at 1 year (Figure 1G), 2 years (Figure 1H), 3 years (Figure 1I), 4 years (Figure 1J), and 7 years (Figure 1K) after treatment initiation. On the last radiograph, it had almost completely disappeared, and root development was completed. No clinical signs or symptoms were present, and root length and thickness had increased. However, the response to the electrical pulp test remained negative.

The patient always showed good adherence and tolerability to the whole process during the treatment and follow up for 7 years without any appointment cancellation.

The protocol used in this case complies with the ESE recommendations [6], except for the concentration of EDTA used.

### Case #2: Revitalisation procedure conducted on an avulsed central incisor at stage 1 root development, following inadequate initial clinical handling and documentation (J.G.L.)

**Figure 2:**
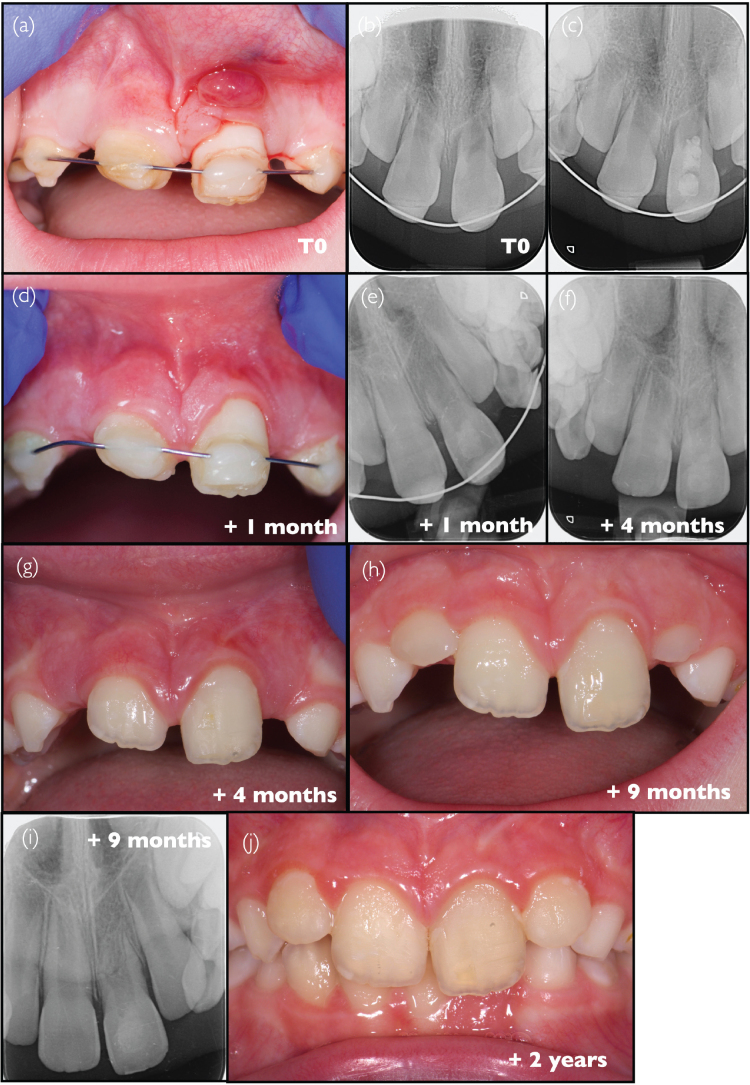

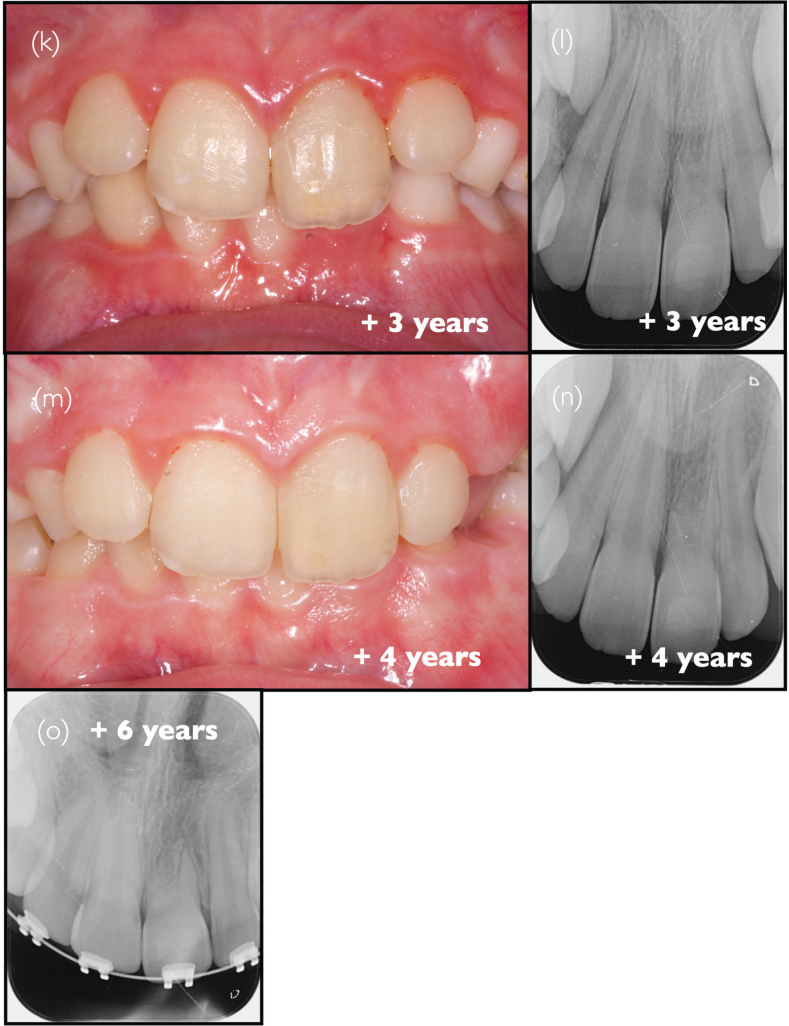
Clinical case #2 (A, B) Pre-operative intraoral photograph and radiograph. (C) Intraoperative radiograph showing the application of a calcium hydroxide agent. (D) Intraoral photograph taken 1 month after the initial visit. (E) Radiograph following the completion of the revitalisation procedure. (F–O) Post-operative follow-up intraoral photographs and radiographs taken periodically from 3 months to 6 years after the procedure.

An 8-year-old female patient with no particular medical history was referred to the endodontic practice (Adult and Child Dentistry, Cliniques Universitaires Saint-Luc, UCLouvain, Brussels, Belgium) by her general dentist for swelling above the upper left central incisor (Figure 2A). The patient had experienced trauma resulting in avulsion of tooth #21, 2 weeks before visiting our clinic. The patient and the referring dentist reported two separate attempts to re-implant/reposition the tooth in two distinct emergency facilities (oral surgeons in hospitals); the second attempt was followed by stabilisation using a rigid splint bonded with resin composite (Figure 2A). The exact conditions of tooth preservation after avulsion, such as time outside the mouth and preservation conditions, could not be documented. This information is nevertheless crucial for determining tooth prognosis, particularly concerning the risk of replacement resorption. A periapical radiograph revealed immature root formation, and although the state of the periapical tissues was unclear, a radiolucent area could be observed along the root, both in the mesial and distal areas, possibly because of repositioning during the emergency operation (Figure 2B). Tooth #21 was painful to palpation and percussion, and was negative to the cold sensitivity test (contrary to tooth #11). The patient did not present any general physical problems. An acute periapical abscess was diagnosed on tooth #21, and a revitalisation procedure was initiated, according to the protocol recommended by the ESE [6]. Although rubber dam could not be placed due to important tooth mobility and lack of retention on the palatal side because of incomplete tooth eruption, any contamination by saliva was avoided by isolation with cotton rolls and constant suction by the dental assistant and made easier by good compliance of the patient throughout the whole procedures. The root canal was accessed and copiously irrigated with 3% NaOCl, followed by the application of calcium hydroxide medication (Pulpdent, USA) into the canal (Figure 2C). The access cavity was temporarily sealed with GIC (Fuji II, GC, Japan). One month later, the pain and swelling had disappeared, and the gingiva had recovered (Figure 2D). The canal was re-accessed and rinsed with EDTA (Largal Ultra, Septodont, France) and finally with saline. The canal was dried, and bleeding was induced by instrumenting beyond the apex with a #30 K-file. When the blood clot reached the cemento-enamel junction level, a haemostatic sponge (Spongostan, Ethicon, USA) was placed on top and covered with tricalcium silicate cement (Biodentine, Septodont, Saint-Maur-des-Fossés, France). The control radiograph after obturation showed the disappearance of the lateral radiolucent areas; however, the interpretation of the state of the periapical area was unclear (Figure 2E). The splint was removed by the general dentist following obturation, and the patient was recalled 3 months later. The examination confirmed a favourable clinical evolution without any signs of infection; progressive eruption of tooth #21 could be observed (Figure 2G). The radiograph indicated a healing tendency of the periapical area (Figure 2F). After 6 months, the eruption of both central incisors had progressed in a seemingly normal manner (Figure 2H). The periapical bone had a normal trabecular pattern around tooth #21, but without closure of the root apex or thickening of the root (Figure 2I). A similar trabecular image could also be observed in the intracanal space. One year later, the incisal edge of tooth #21 was still lower than that of tooth #11 (Figure 2J). Three years after the initial appointment, the clinical condition of tooth #21 was satisfactory (Figure 2K); no signs of inflammation were visible in the periapical area, and the image of a bone-like structure could be observed in the root canal space on the radiograph (Figure 2L). The 4-year recall confirmed the favourable evolution, both clinically (Figure 2M) and radiographically (Figure 2N), both central incisors being almost at the same position on the arch. However, despite the clinical resolution, it must be noted that no further root development could be observed after 4 and 6 years (Figure 2N and O, respectively).

### Case #3: Single appointment revitalisation procedure with large periapical radiolucency due to ancient trauma, in a young adult patient (J.G.L.)

**Figure 3:**
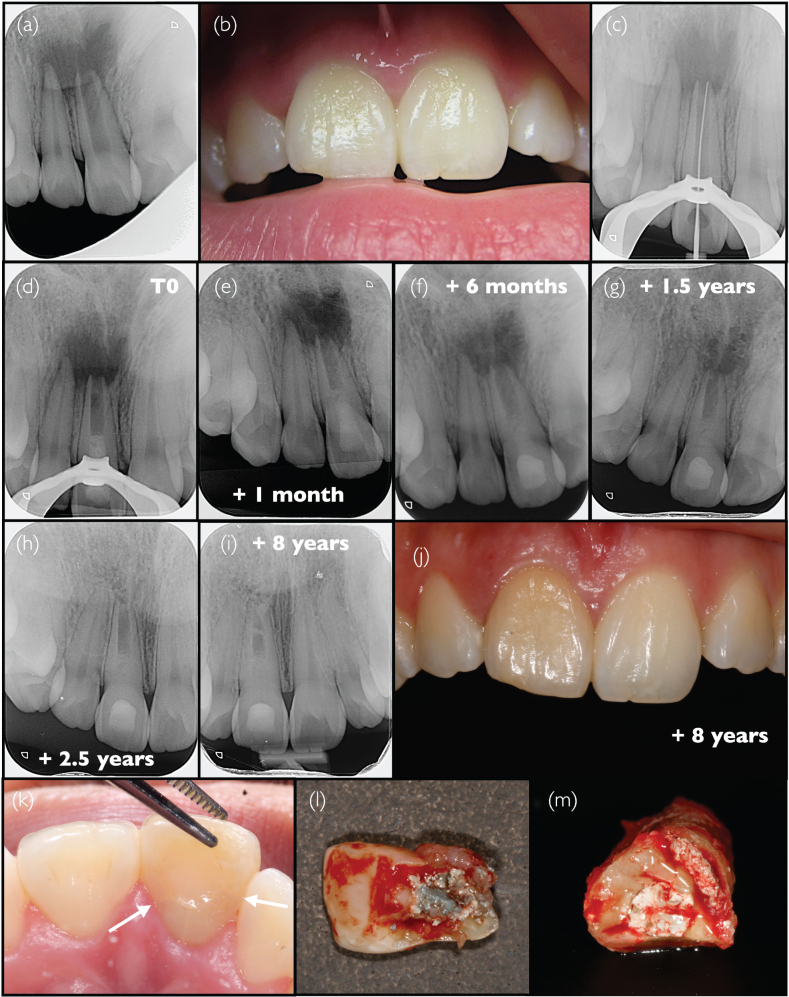
Clinical Case #3 (A) Pre-operative radiograph and (B) intraoral photograph. (C) Intraoperative radiograph showing working length determination and (D) radiograph after completing the revitalisation procedure. (E–J) Post-operative follow-up radiographs taken periodically, from 1 month to 8 years after the procedure. (I) Radiograph obtained during an emergency visit 8 years post-treatment, showing (J) an intraoral photograph and (K) a corono-radicular fracture originating from the palatal side. (L) Image of the extracted crown fragment and (M) the fracture interface.

A 17-year-old female patient without any medical history was transferred to the endodontic practice (Adult and Child Dentistry, Cliniques Universitaires Saint-Luc, Brussels, Belgium) after initial consultation with an oral surgeon regarding a large radiolucent area detected in the apical region of the upper right incisors. The patient had reported in the previous year two episodes of palatal abscess, which were managed by antibiotic prescription. The type and dosage of the antibiotic prescriptions were unknown. According to the report of the oral surgeon, teeth #13-12-11-21 responded positively to cold test, negative to percussion and the patient was totally asymptomatic. The oral surgeon suspected a naso-palatine duct cyst, recommended surgical removal of the periapical lesion under local anaesthesia, followed by an oral pathology examination. Prior to intervention, the surgeon requested root canal treatment of teeth #11 and #12. At the pre-operative endodontic consultation, similar clinical observations to those of the oral surgeon were made, except that tooth #11 was negative to cold test. A periapical radiograph confirmed the presence of a large radiolucency around the apex of tooth #11 (Figure 3A), which presented immature root formation. A slight discoloration could be observed on that tooth as compared to tooth #21 (Figure 3B). The analysis of the clinical findings led to the clinical diagnosis of chronic apical periodontitis, secondary to pulp infection likely because of trauma more than 10 years before. A regenerative endodontic therapy was proposed to the patient instead of the surgery initially planned, and informed consent was obtained. Local anaesthesia without vasoconstrictor (Scandonest 3%; Septodont, Saint-Maur-des-Fossés, France) was administered for this procedure. Given the lack of clinical signs and symptoms, the case was considered as potentially eligible for a single-visit treatment, provided that the canal could be dried after completion of root canal disinfection. This option was discussed with the patient, who provided informed consent for this approach. Consequently, the protocol used in this case deviates from the ESE recommendations [6], because of the absence of a separate disinfection session followed by temporisation with intracanal medication. The treatment, however, took place before the publication of those guidelines. Rubber dam isolation was placed, and the canal was accessed. No signs of vitality could be observed in the canal, confirming the diagnosis. The working length was assessed by a combination of the apex locator (Propex II, Dentsply-Maillefer) and confirmatory periapical radiograph with a file in the root canal (Figure 3C). The canal was abundantly irrigated with a 2.5% NaOCl solution without any instrumentation until no more debris could be seen coming out of the canal under microscope observation. The canal was then dried using paper points to evaluate the presence or absence of exudate or suppuration. Since the canal could be dried appropriately, a final irrigation of 3 min was performed with a commercial 15% EDTA solution (Largal Ultra, Septodont Saint-Maur-des-Fossés, France), followed by a thorough rinsing with NaOCl and drying with paper cone. Bleeding was induced within the canal using a size 35 file 2 mm beyond the apex. When the blood reached 3 mm below the cemento-enamel junction, a piece of haemostatic gelatine-based sponge (Roeko Gelatamp, Coltene) was placed in the root canal. When the sponge was soaked in blood, a fast-setting tricalcium-silicate cement (Biodentine, Septodont Saint-Maur-des-Fossés, France) was placed on top in the whole cavity (Figure 3D). A final radiograph was taken for monitoring purposes (Figure 3E). The patient was reviewed at 1 month for clinical evaluation; the tooth was totally asymptomatic, and the adjacent teeth remained positive to cold test. The tricalcium-silicate cement was then partly removed and covered with a resin-based composite. At the 6-month follow-up, a slight reduction of the periapical radiolucency could already be observed (Figure 3F). One year later, a significant reduction of the radiolucency was observed (Figure 3G) and complete periapical bone healing was achieved two and a half years after completion of the revitalisation procedure (Figure 3H). Apart from the slight and gradual increase in tooth discoloration, the clinical situation remained stable over the years. The patient consistently demonstrated good compliance throughout the entire treatment and follow-up process, and expressed great appreciation for the overall outcome, particularly for the avoidance of surgery. The patient remained asymptomatic at each annual check-up until an emergency visit 8 years after treatment, when she complained of chewing pain. No deterioration of the situation could be observed on the radiograph, with a clear recovery of the periodontal ligament indicated by a continuous lamina dura (Figure 3I). The tooth appeared discoloured (Figure 3J) but no signs of infection could be detected; periodontal probing was normal. However, a crown-root fracture originating from the palatal side was detected (Figure 3K). The level of fracture (Figure 3L) associated with important crown mobility and chewing pain was not compatible with conservative strategies, and an extraction was planned. After extraction, it could be observed that the facture occurred within the Biodentine material (Figure 3M).

### Case #4: Successful revitalisation followed by failure and subsequent success with apical plug placement (M.W)

**Figure 4:**
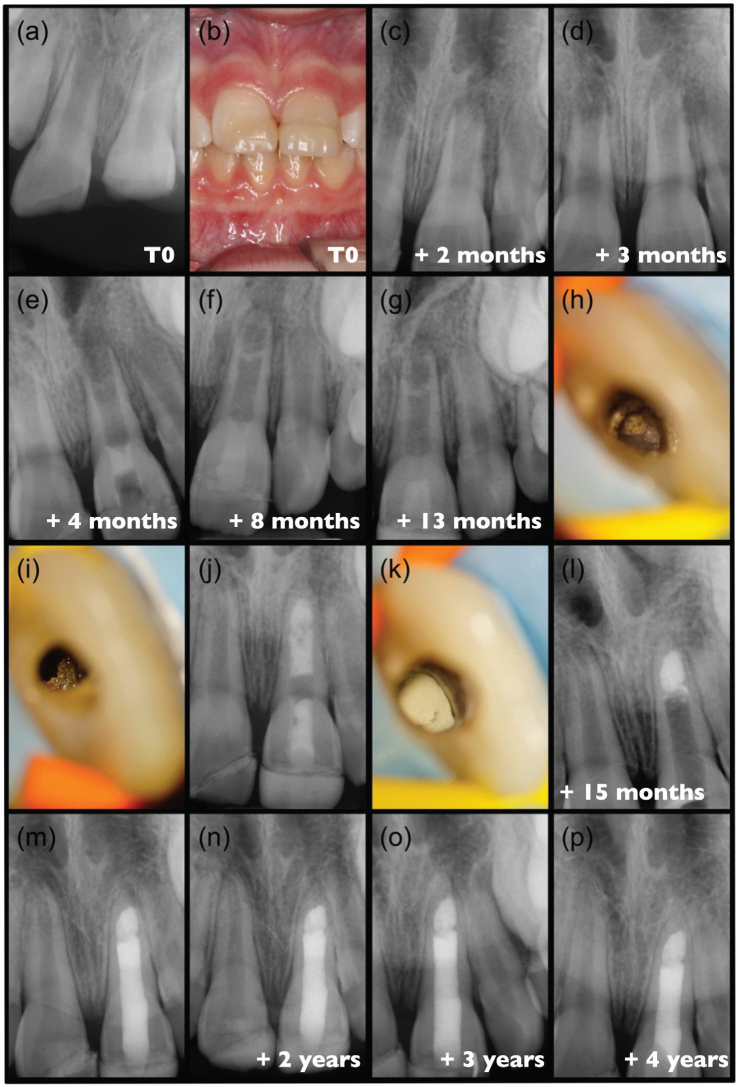
Clinical case #4 (A) pre-partial pulpotomy radiograph. (B) post-pulpotomy intra oral picture. Post-pulpotomy radiograph (C) 2-months and (D) Three-month follow-up. (E) radiograph after completing revitalisation procedure. (F) post-operative radiograph 4 months and (G) 9 months after revitalisation. (H) Irregularly shaped and closed apical foramen, and (I) ectopic calcifications on the canal wall under dental operating microscope. (J) radiograph showed calcium hydroxide applied. (K) Apical plug with mineral trioxide aggregate and (L) post operative radiograph. (m-p) post-operative follow-up radiographs.

An 8-year-old female patient in good general health presented to the Centre for Dental Traumatology at the University Hospital of Regensburg with a dental injury. Examination revealed an enamel-dentine fracture without pulp exposure on tooth #11, and an enamel-dentine fracture with pulp exposure on tooth #21. Both teeth showed no mobility, were sensitive to cold, and radiographs displayed wide open apical foramina and no root fractures (Figure 4A). A partial pulpotomy was performed on tooth #21 using calcium hydroxide, followed by reattachment of the fractured fragments on both teeth (Figure 4B), according to IADT (International Association of Dental Traumatology) guidelines [26]. At the 2-month follow-up, the clinical and radiological findings were normal (Figure 4C). However, 1 month later, tooth #21 developed acute pain, with radiographic evidence of diffuse periapical radiolucency, leading to the diagnosis of acute apical abscess (Figure 4D). The root canal was accessed with rubber dam isolation, disinfected with 1.5% NaOCl and treated with calcium hydroxide for 4 weeks. Finally, 4 months after the trauma, the tooth underwent a revitalisation procedure (Figure 4E) according to the protocol recommended by the ESE [6].

The first follow-up 4 months after revitalisation showed that the tooth was asymptomatic, with no cold sensitivity and evidence of root elongation and apical development on radiographs (Figure 4F). Ectopic intracanal mineralisation was also visible in the apical third. Nine months after revitalisation, the patient again reported symptoms, and radiographs showed a defined periapical radiolucency, corresponding to a diagnosis of exacerbated chronic apical periodontitis (Figure 4G). Thus, despite continued root development in length and thickness, the tooth was accessed under rubber dam isolation after local anaesthesia. Under the operating microscope, vital connective tissue with profuse bleeding was observed in the root canal, together with an irregularly shaped and closed apical foramen (Figure 4H), and mechanically stable ectopic calcifications on the canal walls (Figure 4I). A calcium hydroxide dressing was applied for 8 weeks (Figure 4J).

The tooth remained asymptomatic; therefore, the wide canal was obturated with an apical plug using ProRoot MTA White in the apical third (Figure 4K–L). The remaining root canal was then cleaned, conditioned, and filled with an adhesive restoration using flowable composites placed with an incremental technique under microscopic guidance (Figure 4M). Follow-up examinations at 1, 2 and 3 years post-obturation (Figure 4N–P) showed stable clinical and radiological status with no symptoms. The restorative management was performed by the patient’s general dentist.

### Case #5: Reverse chronology: failed treatment initially managed with imperfect MTA root filling, subsequently resolved through revitalisation (K.G.)

**Figure 5:**
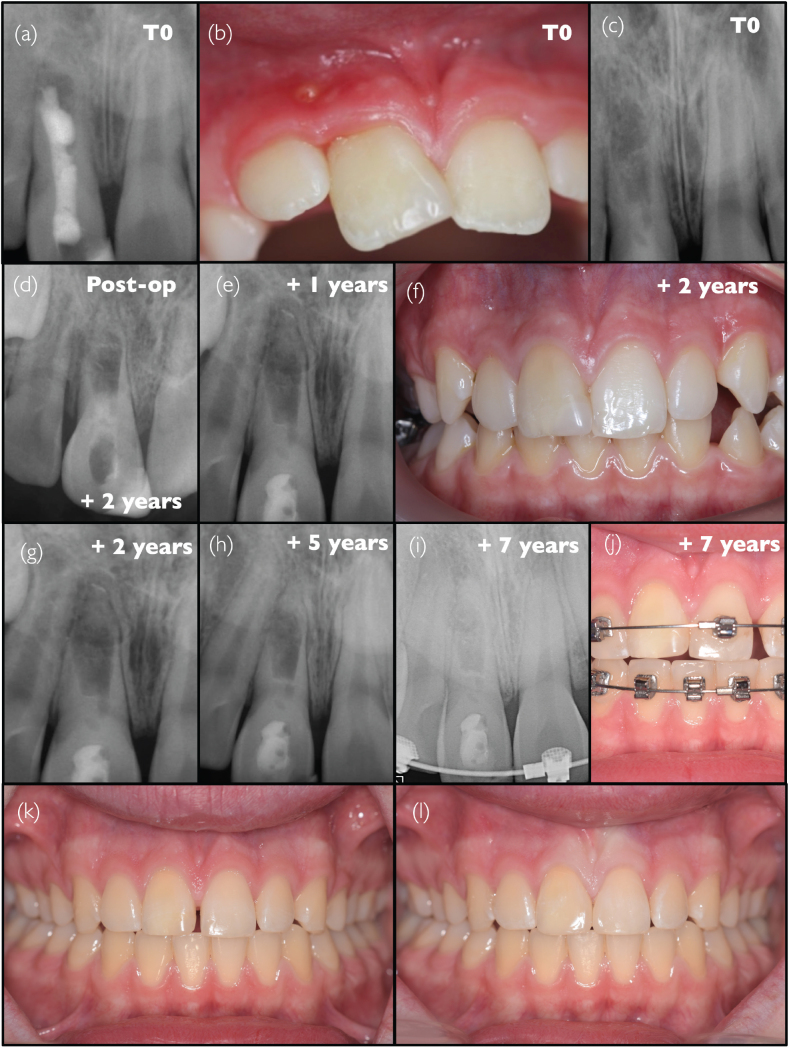
Clinical Case #5 (A) Pre-operative radiograph and (B) intraoral photograph. (C) Intraoperative radiograph taken after the removal of the MTA, followed by the application of a calcium hydroxide agent. (D–J) post-operative follow-up radiographs and intraoral photographs taken periodically up to 7 years. (K–L) post-operative situation following the removal of orthodontic appliances, respectively before and after the closure of gaps using direct composite restorations.

An 8-year old patient with a history of dental trauma of tooth #11 was referred to the University Hospital of Regensburg by his dentist. The boy had suffered from a dislocation injury about 6 months before. Immediate care and follow-ups had been performed in his dentist. Recently, the patient had developed signs and symptoms of inflammation, therefore the tooth was opened and the root canal filled with MTA (Figure 5A). Soon after, a sinus tract became visible (Figure 5B). At the first visit, the tooth was re-opened, the material (MTA), which was loosely packed inside the canal was removed by copious irrigation with saline, the root canal was disinfected by irrigation with 3% NaOCl and filled with calcium hydroxide as intracanal medicament, and the access cavity was filled temporarily (Figure 5C). One week later, the sinus tract had disappeared, and signs and symptoms of inflammation had subsided. Radiographically, the tooth showed a large internal resorption of irregular shape with very thin dentinal walls, in particular on the distal side of the root. In order to avoid further manipulation and choose a minimally invasive procedure, revitalisation was performed, following the protocol published by the ESE [6] (Figure 5D).

Follow-ups with clinical and radiographic examination were performed after 1, 2, and 5 years (Figure 5E–H). The situation remained stable and the tooth in function. Seven years after the revitalisation procedure, orthodontic treatment was planned, and the orthodontist contacted the endodontist for interdisciplinary consultation. It was decided to not put force on tooth #11, but close gaps after treatment using composite (Figure 5I–L). For long-term survival, it is critical that the periodontal ligament is continuous. Whether this was the case would best be evaluated by cone-beam computed tomography (CBCT), but this was declined by the patient and parents. However, this case highlights that revitalisation can also be considered as a second attempt after the failure of the initial strategy, even more so since in this case, the least invasive procedure was sought. It also confirms the potential interest of revitalisation procedures in the management of resorptions, as already mentioned above in Case #1. It further emphasises the question of whether to include these revitalised teeth in orthodontic treatment, a point that was already raised in Case #2.

### Case #6: Apical plug placement chosen over revitalisation based on risk assessment, resulting in complete apical bone healing but persistent gingival defect (J.G.L.)

**Figure 6:**
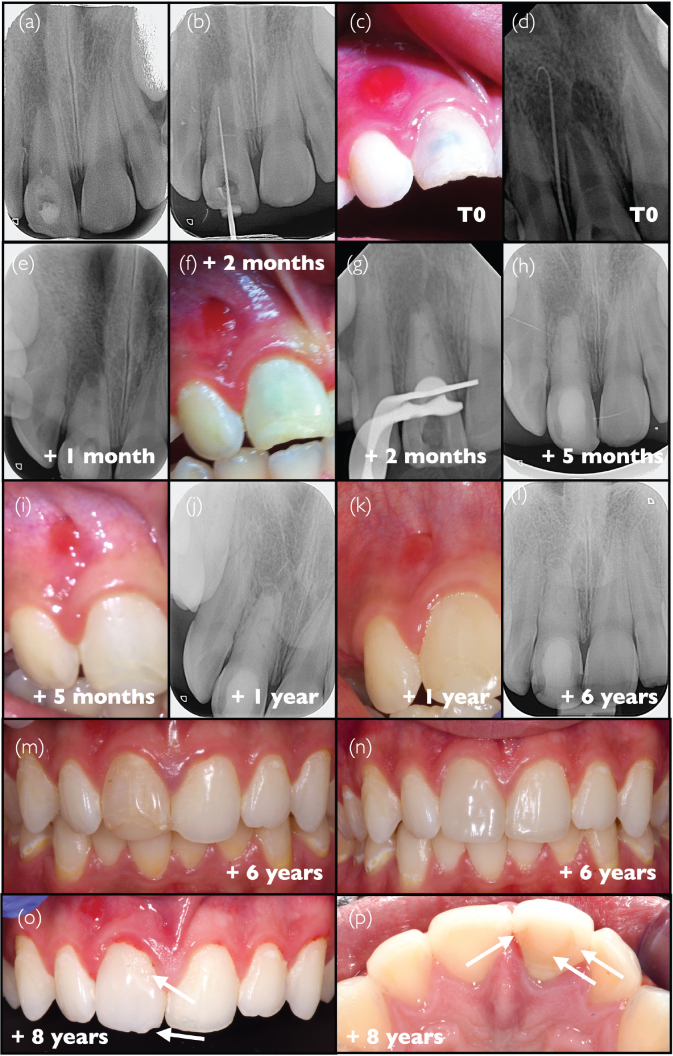
Clinical Case #6 (A, B) Radiographs provided upon referral to the endodontist. (C) Pre-operative intraoral photograph and (D) radiograph showing a gutta-percha point inserted into the sinus tract. (E) Intraoperative radiograph showing the placement of a calcium hydroxide agent, and (F) intraoral photograph. (G) Radiograph taken after completing the apexification procedure. (H) Post-operative follow-up radiograph and intraoral photograph taken periodically over 8 years. (O) and (P) Radiographs taken 8 years later showing composite chipping at both the cervical area and the incisal edge, indicated by white arrows in (O), as well as a crack line on the palatal side, indicated by white arrows in (P).

A 10-year-old male patient in good general health was referred to the endodontic practice (Adult and Child Dentistry, Cliniques Universitaires Saint-Luc, Brussels, Belgium) because of therapeutic difficulties reported by two dentists during the endodontic treatment of tooth #11. Root canal treatment was initiated by a general dentist, followed by a paediatric dentist (Figure 6A and B). The diagnosis leading to the initial canal access was unclear, but according to the anamnesis, it is likely that pulp inflammation was associated with a history of trauma. As in some previous cases, the exact type of dental trauma injury sustained by the patient, leading to the current situation, could not be documented, while it is a major aspect of proper management and prognosis in trauma cases. The endodontic procedure was interrupted by the paediatric dentist because of the patient reporting persistent pain and bleeding upon instrumentation, making completion of the treatment impossible despite several attempts; no rubber dam was used during the procedures, as can be observed on the radiographs. During the first visit with the endodontist, intraoral examination revealed an atypical sinus tract, surrounded by a large, inflamed area, on the gingiva, between the upper-right central and lateral incisors (Figure 6C); it is unknown from the previous dental records whether this was previously observed. Percussion and palpation of the tooth both resulted in pain. There were no periodontal complications, including probing depth or mobility. Teeth #12 and teeth #21 responded positively to the cold test, while teeth #11 was negative. A radiograph was taken with a gutta percha cone in the sinus tract (Figure 6D), and a radiolucent area was observed around the apex of teeth #11, compared to teeth #21. No specific general or family medical histories were recorded. Based on these clinical findings, a diagnosis of chronic apical periodontitis with sinus tract was made, which was likely caused by an infection through coronal cracks at an immature stage of root development. The root canal was re-accessed under local anaesthesia and under rubber dam, and abundant bleeding was observed upon removal of the temporary obturation. The canal was profusely irrigated with 3% NaOCl, and the working length was determined radiographically using a #30 K-file. A calcium hydroxide medication (Pulpdent, USA) was placed into the canal and temporarily sealed with GIC (Fuji IX, GC, Japan). At the next appointment, 1 month later, the patient was asymptomatic, but the atypical sinus tract remained visible. Instrumentation to working length and irrigation with 3% NaOCl were repeated under rubber dam, and a mixture of pure calcium hydroxide and water was placed in the canal (Figure 6E). Another month later, the sinus tract remained present despite an obvious improvement of the clinical situation (Figure 6F), probing of the sinus tract was however no longer possible. The canal was re-accessed under rubber dam and could be dried. However, due to the persistence of the gingival lesion combined with a stage of root development >2, it was decided that the tooth would rather benefit from the placement of an apical plug with a hydraulic tricalcium silicate cement (Biodentine, Septodont, Saint-Maur-des-Fossés, France) instead of a revitalisation procedure (Figure 6G). This happens to be in accordance with the recent S3-level guidelines [5]. Five months later, the periapical radiograph revealed a reduction in periapical radiolucency (Figure 6H); an additional reduction of the gingival lesion was observed, although it remained present (Figure 6I). Owing to the positive evolution, monitoring of the tooth was continued. After 7 months, the periapical bone appeared fully healed on the control radiograph (Figure 6J), while minimal redness was still visible on the gingiva (Figure 6K). Six years after the initial visit, the radiograph indicated full healing with normal structure of both the root and the apical tissues (Figure 6L); however, the small mucosal redness persisted (Figure 6M). In the absence of symptoms and based on additional advice from a periodontologist, additional annual monitoring of the lesion will be continued, and the patient requested the placement of a composite veneer (Figure 6N). Unfortunately, despite being, until then, very compliant with the treatment, the patient did not attend the yearly check-up, and only returned 2 years later, requesting an emergency appointment after hearing a crack sound. Clinical examination revealed a non-restorable root fracture (Figure 6O and P).

## Discussion

The case series presented above highlights the diversity of cases that may be eligible for revitalisation procedures and underscores the numerous challenges that must be addressed to better understand the potential and limitations of this treatment strategy, as compared to the alternatives.

These challenges should be considered through the lens of the various ways of defining the outcomes of revitalisation procedures, which have been categorised into three different levels: scientist-, clinician-, and patient-based outcomes [16].

During the development of the ESE S3-level guidelines, tooth survival was identified as the most critical patient-centred outcome, in combination with the clinical resolution of periapical diseases [27].

However, in the context of immature teeth, other functions may also be important, such as the ability to undergo orthodontic movement, or the management of root resorption. As highlighted in cases #1, #2, and #5, it remains uncertain whether such teeth can be safely and effectively moved during orthodontic treatment, which may be needed years after revitalisation. A recent cohort study found no significant impact of orthodontic treatment on traumatised immature incisors, including revitalised cases [28]. However, further clinical data are needed to confirm this, particularly regarding resorption. In this context, recent reports suggest that revitalisation procedures may offer potential benefits [29, 30], presenting a promising future approach for managing these complex cases.

Clinician-based outcomes include patient-based outcomes, while also incorporating positive responses to pulp sensitivity tests, as well as radiographic indicators of periapical bone healing and continued root development (in terms of root length and width). Finally, scientist-based outcomes require histologic evidence of the complete regeneration of the pulp-dentin complex, rather than replacement by bone-like tissues, fibrous connective tissues, or cementum, which would be classified as repair rather than true regeneration.

Based on these definitions, this work aims to outline the main areas where biomaterials research can make significant contributions. The challenges faced by researchers, in particular in the field of biomaterials, can be broadly categorised into biological aspects aimed at improving treatment success and mechanical challenges focussed on maximising tooth survival.

### Treatment success and related biological challenges

Endodontic treatment success is classically defined for standard root canal treatments by the absence of clinical signs, such as swelling or drainage, and symptoms, such as pain or discomfort, along with the absence or complete resolution of periapical radiolucencies [31]. In applying similar criteria to revitalisation procedures, reported success rates are high – typically 75% or higher [11, 17, 23, 32–34] – comparable to the range of success rate values reported for root canal treatments in mature teeth [35] (below 5 years follow-up). Despite these promising results, it should be noted that the reported success rates of revitalisation therapies are based on data with relatively short follow-up periods, mostly under 2 years. According to the few available studies evaluating the probability of root canal treatment success over long periods of time (20 years or more), short-term success rates are high, but progressively decrease over time [31, 36]. This trend is likely to be expected for revitalisation cases as well. Therefore, much longer follow-up is needed for these therapies, which is precisely the contribution of the cases presented in the present paper, with follow-up ranging from 4 to 8 years. Overall, the level of evidence of revitalisation procedures has been described as low [11, 33].

To our knowledge, only a few clinical trials to date have compared revitalisation with a control procedure, such as the classical apexification with calcium hydroxide or the placement of an apical plug consisting of a hydraulic calcium silicate cement [34, 37–42]. Historically, apexification was the only available option and consisted of using repeated intra-canal applications of calcium hydroxide to induce closure of the open apex to facilitate a more favourable condition for root canal filling with gutta percha [43, 44]. Currently, this technique is no longer recommended. Instead, the placement of an apical plug using hydraulic calcium-silicate cement in a single visit is preferred as the first option [7, 9]. Revitalisation is considered a viable alternative, particularly favoured in cases of very immature stages of root development [5]. This differentiation, based on the stage of root development, retrospectively supports the therapeutic decisions made, for example, in cases #2 and #6.

None of the studies including one of these control procedures have identified a significant difference with revitalisation as regards periapical healing [34, 37–42]. Some differences were, nevertheless, highlighted in favour of the revitalisation strategy, but only for certain purely clinician-based outcomes mentioned earlier, such as root length or thickness, apical diameter or pulp sensitivity, which are not included in patient-based outcomes. Interestingly, cases #4 and #5 pointed out that both procedures are not necessarily mutually exclusive but can serve as alternative treatment options, in cases of treatment failure.

Overall, the degree of success for these clinician-based outcomes has been described as inconsistent [11, 17, 23, 34, 45]. This variability likely arises from the low prevalence of these cases overall combined with the numerous variations of the clinical protocols over the years [46], resulting in the insufficient knowledge of the effect of each treatment parameter on treatment outcome. While some therapeutic strategies may yield positive effects, they can still differ significantly in key aspects, such as the number of treatment sessions (case #3) or the concentration of EDTA used (case #1). As mentioned by Galler et al. in the ESE position statements [6], the recommendations certainly need to be updated in the light of new evidence as it emerges. However, it is important to communicate to clinicians the importance of applying the current consensus procedures until the next update. For comparative purposes, it is indeed more reasonable for clinical practice to evolve in stages rather than continuously, as it allows to properly evaluate the impact of each procedural change. The availability of clinical guidelines and standardised protocols [6–8] is therefore very valuable, since adherence to these guidelines and protocols helps reduce the variability in the reported studies and cases, thereby enabling more robust comparisons. Disinfection strategies have drastically changed since the introduction of revitalisation procedures, with various types, concentrations, and sequences of irrigants and medications [15, 46], as illustrated in the present work by the diversity of the protocols in the different cases. Root canal disinfection strategy has been demonstrated to be of major importance both clinically on the success of the procedure [47], and *in vitro* on stem cell fate [48, 49]. However, these various protocols have not translated into clear differences at the clinical level, as demonstrated by a recent randomised clinical trial (RCT) that failed to show the superiority of one disinfection strategy over another (calcium hydroxide vs. triple antibiotic paste) [50]. In any case, there is a clear need to improve the understanding of the interactions between bacteria and the cells migrating into the canals [49, 51].

Similarly, while guidelines recommend performing revitalisation in two sessions with intracanal medication (unlike in case #3), evidence to strictly support this remains limited. A randomised trial indeed suggested a lower success rate for the single-appointment approach (33% compared to 71% for two sessions), but fell short of reaching the necessary sample size at inclusion and the 12-month follow-up, and should therefore be interpreted with caution [52].

Despite the aforementioned lack of significant differences in patient-centred success criteria, the available clinical studies have allowed us to make some interesting observations that could offer valuable insights for future directions in the field. In a 12-month RCT comparing classical apexification and revitalisation procedures [34], root thickness and length were found to be significantly greater with revitalisation. For this reason, it has been suggested [5, 17, 20] that revitalisation procedures should be considered, particularly for cases with severely deficient root development, such as case #2 or case #4, or when the root canal has been significantly enlarged because of resorptive processes, as in case #5. This approach is indeed generally simpler than placing an apical plug in such situations.

Moreover, aetiology was shown to impact the outcomes, with cases of dens evaginatus (like case #1) being associated with significantly better results compared to trauma cases (like cases #2 to #6) [53].

Traumatic damage to Hertwig’s epithelial root sheath, which plays a key role in the reciprocal interaction with neural crest-derived cells during root formation [54], has been suggested as a major contributing factor to the reduced success of revitalisation procedures [55–57]. In addition, such damage may lead to other adverse outcomes for the patient, including root resorption, ankylosis, or infra-position, depending on the severity of the trauma itself, as well as on extraoral time and storage conditions of the tooth. While these questions go well beyond the scope of the present paper, it is important to underline that the type and severity of traumatic injury are likely to have a greater impact on the long-term fate of the tooth than the decision to attempt a revitalisation procedure or place an apical plug of a hydraulic calcium silicate cement. Thus, it is crucial that future research contributes to understanding which types of injuries are suitable for revitalisation and which are not, emphasising the need for accurate documentation of the specific conditions of the trauma.

In order to improve the outcomes of revitalisation, attempts were made to include additional growth factors into the scaffold or the blood clot itself, either without additional benefits as compared to the standard blood clot induction [41], or with marginal ones regarding some parameters [42]. Similarly, the additional use of a specific collagen membrane was only able to improve certain non-patient-centred parameters and the convenience of use [58]. The use of platelet-rich fibrin or plasma have been suggested as another possible improvement to the procedure, but studies have shown either no positive effect compared to a blood clot [40, 59] or only minor yet statistically significant improvements in root thickness [39]. Platelet-rich fibrin and concentrated growth factor platelet scaffolds were also directly compared, without any superiority being identified between the two [38]. A recent systematic review and meta-analysis concluded to the lack of significant impact of the use of various scaffolds on the therapeutic efficacy of revitalisation procedures [60].

Scientist-based outcomes have not been extensively addressed yet, since, as mentioned earlier, no consistent evidence of actual regeneration of the pulp-dentin complex has been demonstrated. The currently observed root canal obliteration by tissues other than dentin and pulp may be due to the absence of an appropriate exogenous scaffold to adequately guide cells and direct their fate. Root canal obliteration is also observed in spontaneous revascularisation in trauma cases, as documented in case-reports [61] and in a systematic review [62]. This has also been described in the context of tooth auto-transplantation, referring to the presence of a ‘nature-engineered’ pulp scaffold remaining present [63]. Along this line, the use of decellularized dental pulp scaffolds were also suggested, and have shown interesting results, with the advantage of closely mimicking the original stem cell niche [64]. Hence, further research is needed to better understand the molecular mechanisms regulating odontogenesis and pulp-dentin complex healing in such situations. This will allow new developments in scaffold materials, which are essential to better guide cells towards the regeneration of a newly formed pulp tissue, producing dentin.

Some perspectives hold promise in this regard. For example, bioactive scaffold material have been introduced in regenerative medicine, possessing features such as the controlled release of growth factors [65], 3D structures allowing fine-tuning of key properties to promote actual tissue regeneration [66], or antibacterial activity such as an antibiotic-laden scaffold [67] or others [68] to further contribute to the necessary disinfection of the root canal space. Other very innovative and integrative approaches have also highlighted the need to consider both the infectious and inflammatory aspects of the regenerative challenges, with the development of membranes promoting symbiosis, preventing detrimental inflammatory responses, and improving cellular integration [69].

As a near-future perspective, some significant advances have been made in the field of biomaterials for pulp therapy, which could be highly beneficial in the context of revitalisation. For example, lithium-containing glass fillers have been shown to enhance dental pulp cell functions and induce reparative dentin formation through the activation of the canonical Wnt/β signalling pathway [70]. Another interesting perspective is the identification of a peptide identified after degradation of organic dentin matrix components by matrix metalloproteinase-20, that exerts anti-inflammatory activity and thereby promotes tertiary dentin formation [71].

The question of the material used over the blood clot or scaffold also needs to be addressed. In the first case report of revitalisation published, the material used was reported to be GIC [72]. Following this initial report, hydraulic calcium silicate cements became the predominant materials applied in these procedures [73], and they were later incorporated into the standard protocol recommended by the AAE [74]. Over 85% of revitalisation studies conducted between 1993 and 2014 utilised MTA [46], likely because of its widespread availability. Currently, materials such as ProRoot MTA and Biodentine are the most commonly used materials for ‘capping’ blood clots in the canal during revitalisation procedures, largely because of their high biocompatibility and effective sealing properties, and indeed, these materials were used in all cases in the present case series. In recent years, a variety of newer, generic materials have been developed as alternatives, with performance reported to be comparable to MTA [75]. This would need to be confirmed for the specific application of revitalisation. At the moment, there is no clear consensus on the most optimal material for this application. When calcium silicate cements are applied, they are expected to exert a biological effect on the blood clot containing stem cells. In pulp biology, they have been demonstrated, such as calcium hydroxide or EDTA, to have the ability to induce growth factor release from the dentin, for example TGF-β [76–78], which can then promote repair-associated events within the pulp [79], by influencing pulp progenitor cell fate [80]. Besides this indirect effect, MTA was also shown to directly stimulate odontogenic differentiation of pulp cells, particularly in uninduced cells [81], which could be of importance in the situation of the uninduced cells present in the blood clot. However, there is presently no strong evidence to determine whether calcium silicate cements effectively promote the regeneration or repair processes by directing the fate of the cells within the blood clot, which deserves to be further investigated. Moreover, translational and clinical studies specifically investigating these aspects remain limited [82].

Finally, an important clinical consideration with regards to these procedures is coronal discoloration, which can be a significant concern for patients. ProRoot MTA^®^ has been associated with greater discoloration in most reports, whereas Biodentine has demonstrated better tooth colour stability [83–85]. The discoloration from MTA is often attributed to the presence of bismuth oxide and iron oxide, as well as potential blood contamination in the pulp and the use of sodium hypochlorite during irrigation [75, 86]. Newer calcium silicate materials that use zirconium oxide as an alternative to bismuth oxide have shown reduced tooth discoloration [87]. This is clinically very significant as it impacts aesthetics, which is of great importance for patients, particularly in trauma cases which affect primarily anterior teeth. While none of the anterior cases treated with Biodentine (cases #2, 3, 4 and 5) presented any grey discoloration, a yellowish discoloration still appeared over time.

To conclude this section, it is important to note that, although some variables were demonstrated to have statistically significant impact on some specific outcomes of the revitalisation procedures, it remains unclear whether an increase of about a millimetre in root length and a few tenths of a millimetre in root thickness holds any clinically meaningful, patient-based significance. Regarding the most crucial patient-based outcome – tooth survival – there is currently no robust evidence to support this, as will be discussed in the next section.

### Tooth survival and related mechanical challenges

Preserving a functional, endodontically treated tooth is a top priority for patients, second only to trust and communication with their practitioner in their expectations [88]. Therefore, maximising tooth longevity should probably be considered as the key patient-centred outcome in the treatment of immature necrotic teeth. It is regrettable that patient-centred outcomes have been reported in less than 15% of apexification studies, compared to clinician-centred outcomes, which were reported in over 85% of studies [89]. The significance of prioritising patient-centred outcomes over clinician-based ones is clearly demonstrated in cases #3 and #6, where cervical root fractures occurred 8 years post-treatment, despite achieving biologically favourable results. This further emphasises the paramount importance of tooth preservation as the most critical clinical outcome [27]. Case #6 highlights the challenge of adequately disinfecting the root canal in immature teeth to achieve periapical healing without compromising tooth structure, as evidenced by incomplete gingival healing despite full bone resolution [90].

Therefore, besides significant biological implications, pulp loss can compromise the long-term survival of immature teeth because of reduced mechanical strength. This problem is particularly severe for teeth in the early stages of root development, which have been shown in both classical *in vitro* studies [91] and clinical studies [92, 93] to be more predisposed to fracture due to their short roots and thin dentin walls. Pulp regeneration could help to increase root length and dentin wall thickness, thereby potentially contributing to increased long-term tooth survival by improving mechanical strength. For this reason, as mentioned above, it was proposed that revitalisation procedures would be more beneficial for immature teeth with pulp necrosis at early stages of root development, typically stages 1 and 2 [5], possibly stage 3 [17]. In contrast, for teeth where root formation is nearly complete (stage 4), the apical plug technique has been described as a more suitable approach [17].

Nevertheless, the promise of pulp-dentin regeneration offered by the concept of regenerative endodontics to complete root development and strengthen teeth explains why this strategy rapidly gained popularity among endodontists after its clinical introduction. This reasoning was elegantly demonstrated in an animal study [94], where teeth that exhibited a relative increase in root length – double that of the controls – also showed more than a twofold increase in fracture resistance (rising from approximately 100 N to over 250 N). However, on the one hand, as mentioned earlier, root growth in terms of lengthening and thickening is unpredictable and cannot be guaranteed in all cases, given the large diversity of clinical situations. On the other hand, even when further root development does occur, systematic reviews and meta-analyses have not been able to demonstrate a significant increase in tooth survival compared to control treatments [11, 18, 20]. However, as mentioned earlier, these studies are based on original clinical data that are mostly limited to 12-month follow-up periods, and only rarely extend up to 2 years. At such short-term follow-ups, the pooled survival rates are very high, exceeding 95% [11, 18]. When assessing tooth survival, failure occurs on a different timescale, as seen in cases #3 and #6. As with success, but even more critically, long-term data are therefore essential.

This point is particularly well illustrated by a recent longitudinal study on the outcome of endodontic treatment of immature necrotic incisors. This study did not include revascularisation but was limited to apexification, either with repeated calcium hydroxide application or a MTA apical plug, with mature teeth treated using conventional root canal treatment serving as the control [93]. The control teeth demonstrated a survival probability of approximately 80% at 20 years, in line with the values reported for anterior teeth in two recent long-term studies on mature teeth [31, 95]. Before 2 years postoperatively, almost no differences in survival were observed between the various prognostic variables considered for apexified tooth survival, with their survival probability being in the same range as that of mature teeth, as confirmed by another recent retrospective cohort study on apexification [96]. However, as the follow-up period extends, significant differences begin to emerge, particularly after 5 years and beyond. The vari-ables that significantly and negatively affected tooth survival were calcium hydroxide apexification (hazard ratio [HR] = 13.4 as compared to control), avulsion (HR = 5.6 as compared to control), and increasing size of periapical radiolucency (HR = 2 to 4 as compared to control). The median survival times reported for each treatment strategy were 10 years for calcium hydroxide apexification, 16.1 years for MTA apical plug, and 20 years for standard root canal treatment in mature teeth (control). The median survival times corresponding to the type of traumatic injury were 15.5 years for luxations, 12.5 years for intrusions, and 6.8 years for avulsions [93]. Considering these numbers, it becomes obvious that the lack of significant differences observed at short term between revitalisation and apexification procedures is to be considered with caution. Although some case reports have documented root fractures occurring in the short term (2 years) [97, 98] and even very short term (<1 month) [99], evaluating the primary patient-based outcome for the treatment of immature necrotic teeth requires follow-ups of at least 5 years to offer meaningful guidance on which treatment strategy to recommend.

While unrestorable root fracture has been consistently reported as the main reason for extraction of mature endodontically-treated teeth [31, 100, 101], the risk of extraction because of fracture appears even more critical in immature necrotic teeth, with its frequency increasing as the stage of root development decreases [92, 93]. Therefore, restorative aspects aimed at reducing the risk of fracture of immature necrotic teeth should be considered a key factor in both regenerative endodontic treatments and traditional approaches.

From a restorative standpoint, the best contribution to long-term stability is to anchor an adhesive restoration as deeply as possible in the root canal, which can typically be achieved with apical plug strategies [102–104]. The use of fibre posts, in particular, has shown some promise for tooth reinforcement *in vitro* for this specific application. However, the authors of a recent systematic review advised caution in interpreting these results due to an unclear or high risk of bias [105]. Further well-conducted research on the topic is therefore needed.

Unlike in case of apical plug placement, a successful revitalisation procedure results in the root canal being filled with tissue up to the cemento-enamel junction, which prevents the deep placement of an adhesive restoration. However, it has been reported that simply covering the blood clot and capping material with an adhesive restoration increased fracture resistance underlining the important role of the restoration after revitalisation [106, 107].

Hence, both root growth and deep adhesive restoration have the potential to positively impact the longevity of endodontically treated immature teeth. As previously mentioned, clinical investigations have so far failed to demonstrate the superiority of any specific strategy in terms of tooth survival [11, 18, 20]. Nevertheless, further efforts should be directed towards developing restorative approaches aimed at preventing this specific type of failure, namely cervical root fracture. In this context, finite element analysis (FEA) has proved a useful tool to compare the mechanical behaviour of different treatment scenarios. The FEA is a computational method used to simulate and analyse physical systems and structures, such as the hard tissues of a tooth and restorative materials. It allows the study of how these structures respond to various physical effects, such as mechanical loading, as they occur in the function of teeth. Similar to *in vitro* studies, FEA analyses emphasised the importance of adequate restoration after both revitalisation and apical plug therapy, which reduces stress in the coronal tooth area [108]. Extending the adhesive restoration into the root canal, as suggested above, can further decrease mechanical stress in the mid-root canal [102], where stress tends to be high [109]. However, FEA models including surrounding tissues also show high mechanical stress in the apical region, which can be mitigated by root maturation [110]. Regardless of the type of tissue deposited after revitalisation treatment (bone, cementum, or dentin) stress can be more effectively distributed across the larger surface area of a mature, fracture-resistant tooth root [110], thereby possibly increasing tooth longevity over the long-term.

## Conclusion

The endodontic treatment of immature teeth with pulp necrosis is a complex yet captivating topic, as demonstrated by various atypical clinical cases. Two main categories of challenges have been identified.

On one hand, biological challenges need to be addressed to improve not only patient-centred outcomes but also clinician- and scientist-centred outcomes. While the latter two may seem more removed from patient concerns, they are crucial for deepening the understanding of this treatment strategy and optimising the procedure. On the other hand, mechanical challenges were identified as particularly significant for improving the main patient-centred outcome: reducing the incidence of cervical root fractures, thereby enhancing tooth longevity.

Meeting these challenges requires close collaboration between both researchers and clinicians, to establish guidelines, evaluate and understand treatment outcomes, and update guidelines accordingly. The insights and discussions arising from this case series, combined with both classic and recent literature will hopefully contribute to facilitate mutual understanding and collaboration, to allow further progress in the field of endodontic treatment of immature teeth with pulp necrosis, and to expand the use of regenerative dental medicine.
